# Comparative Analysis of In-Match Physical Requirements Across National and International Competitive Contexts in Cerebral Palsy Football

**DOI:** 10.3390/s25123834

**Published:** 2025-06-19

**Authors:** Juan Francisco Maggiolo, Juan José García-Hernández, Manuel Moya-Ramón, Iván Peña-González

**Affiliations:** 1 Sports Research Centre (Department of Sport Sciences), Miguel Hernández University of Elche, 03202 Alicante, Spain; fran.maggiolo@goumh.umh.es (J.F.M.); ipena@umh.es (I.P.-G.); 2 Spanish Federation of Sports for People with Cerebral Palsy and Acquired Brain Injury (FEDPC), 28008 Madrid, Spain; juanjo.gh@fundacionsegundaparte.org; 3 Faculty of Health Sciences, Exercise and Sport Sciences, University of Francisco de Vitoria, 28223 Madrid, Spain; 4 Segunda Parte Foundation, 28034 Madrid, Spain

**Keywords:** CP football, player load, team para-sport, neurological impairments

## Abstract

**Highlights:**

**What are the main findings?**

- This is the first study to demonstrate that physical and technical demands in cerebral palsy (CP) football increase substantially with competitive level, with international matches (World Cup) imposing up to three times greater workload compared to national-level phases.
- Players exhibited significantly higher sprinting, accelerations, decelerations, and ball contact rates in international contexts, even when the same athletes competed across different levels.

**What is the implication of the main finding?**

- These findings underscore the need for tailored training programs that replicate the intensity and complexity of elite CP football competition.
- The use of wearable inertial sensors, as applied in this study, proves to be an essential tool for accurately monitoring match and training loads, enabling performance optimization and individualized training strategies based on competitive context.

**Abstract:**

This study aimed to compare in-match physical and technical requirements of cerebral palsy (CP) football players across different national and international competitive contexts. A total of 79 male outfield players participated in 62 official matches across 3 competitive phases of the Spanish National CP Football League (Regular Phase, Consolation Phase, and Playoffs) and the IFCPF World Cup. Inertial measurement units (IMUs) were used to record locomotor and technical variables during each match. A subset of 10 players was tracked across all phases. Physical demands were normalized per minute of play and analyzed using one-way and repeated-measures ANOVAs. Results revealed that physical requirements during the World Cup were up to three times higher than during national-level matches, with significantly greater maximum velocities, high-intensity distances, and frequencies of accelerations and decelerations (*p* < 0.001, ηp^2^ > 0.40). Playoffs also imposed significantly greater physical requirements compared to Regular and Consolation Phases. International matches showed a markedly higher number of ball contacts, indicating increased technical involvement. These patterns were consistent in both the full sample and the longitudinal subsample, suggesting that competitive level—rather than player characteristics alone— strongly modulates physical output during the competition. These findings underscore the need for context-specific training and load management strategies to prepare athletes for the elevated demands of high-level CP football competition.

## 1. Introduction

Cerebral Palsy (CP) football is a para-sport modality governed by the International Federation of Cerebral Palsy Football (IFCPF) and designed for athletes with neurological conditions such as CP, traumatic brain injury, or stroke. These conditions may cause impairments in muscle coordination, balance, and motor control, which influence the way players move, react, and interact with the ball during competition. The sport follows modified FIFA rules and is played in a 7-a-side format, with smaller pitches, no offside rule, and shorter match durations [1]. Importantly, all players must be classified into one of three functional classes—FT1, FT2, or FT3—based on the severity and type of motor impairment, ensuring fair and inclusive participation [2]. CP football was part of the Paralympic Games until Rio 2016 and continues to be a key competition on the international para-sport calendar.

Despite the growing popularity and professionalization of CP football, the scientific literature on the physical and technical requirements of the sport remains limited, especially when compared to the extensive body of work in able-bodied (mainstream) football. Understanding the physical and technical requirements experienced by players during match play is crucial for designing evidence-based training programs, optimizing performance, preventing injuries, and informing classification procedures. In recent years, the development and refinement of wearable inertial measurement units (IMUs) has significantly advanced the monitoring of physical performance in team sports. These microelectromechanical systems (MEMS), which combine accelerometers, gyroscopes, and magnetometers, have made it possible to accurately quantify a wide range of locomotor variables—such as total distance covered, velocities across different intensity zones, and the frequency and magnitude of accelerations and decelerations [3]. Widely validated in mainstream football, IMUs have become a standard tool for performance analysis, training load management, and injury risk reduction. Their portability and independence from fixed infrastructure make them especially valuable in para-sport contexts, where competition and training environments often vary in structure and accessibility.

In CP football, the use of IMUs has expanded considerably, offering reliable insights not only during competition [4,5,6] but also in training environments [7]. These devices have proven effective in characterizing the physical requirements experienced by players during official matches, with studies identifying variations based on functional classification and contextual factors such as opposition level or match outcome. As such, wearable IMU technology not only enhances the precision of match analysis but also supports individualized training prescription and long-term player development in para-sport. Previous studies that have used this technology in CP football have shown that physical requirements vary depending on players’ functional classification and contextual factors. For example, players with lower levels of impairment (i.e., FT3) consistently demonstrate greater match-running performance than their peers with more severe impairments, covering more distance at high-intensity running and sprinting speeds, and performing more frequent short-duration, high-intensity actions such as accelerations and decelerations during competition [8]. In a full-season analysis of the Spanish National League, FT3 players were also found to outperform FT2 and FT1 players in key workload variables, including total distance, high-speed running, and impact load [6]. These differences demonstrate the practical relevance of tailoring training according to players’ sport class to address their functional heterogeneity. Similarly, Yanci et al. [4] reported that CP footballers cover substantially less distances at high speeds and perform fewer high-intensity neuromuscular actions than players in mainstream football, highlighting the influence of impairment on match activity profiles [4]. Furthermore, contextual conditions such as altitude [9] or match competitiveness and team ranking [5] also modulate the CP football player’s physical requirements, suggesting that both intrinsic (e.g., sport class) and extrinsic (e.g., competitive contexts) factors must be considered when evaluating or planning training loads in this para-sport.

In team sports, a substantial body of evidence indicates that the competitive context—defined by factors such as tournament stage, match importance, opponent quality, and tactical complexity—has a direct impact on player behavior and physical output. In mainstream football, some studies have shown that players in top-tier competitions (e.g., Premier League) showed higher in-match physical requirements and engage in more frequent and complex technical actions compared to those in lower divisions [10,11]. However, other findings suggest that players in lower levels may cover longer total distances due to less organized tactical structures [12], highlighting the complexity of interpreting match demands solely through the lens of competition level. Furthermore, contextual variables such as player position, scoreline, or time remaining have been shown to mediate player’s in-match physical requirements independently of competition level.

In the context of CP football, preliminary evidence has begun to reveal similar patterns to those observed in mainstream football regarding how contextual variables modulate player physical requirements in competition. For instance, international-level players have been shown to possess greater physical fitness and technical skill compared to their national-level counterparts [13]. Additionally, a positive relationship has been established between physical performance in field-based tests and in-match locomotor requirements among CP football players [14]. Expanding upon this, recent research has provided deeper insight into how contextual variables influence in-match physical requirements in international competitions. Henríquez et al. [5] demonstrated that physical responses in CP football are significantly affected by contextual factors such as team ranking, quality of opposition, and match outcome. Specifically, players from top-ranked teams exhibited greater physical requirements when facing similarly ranked opponents, particularly in sprint distance and acceleration/deceleration metrics. Conversely, bottom-ranked teams showed increased running requirements and short-term actions when playing against teams of equivalent level. These findings highlight that beyond individual player capacity or impairment level, competitive context plays a crucial role in shaping physical output. Furthermore, this evidence reinforces the value of contextualized performance analysis for optimizing training load, preparing for elite competition, and informing classification processes in CP football.

While prior studies have examined contextual influences on match demands in CP football, none have directly compared physical and technical performance across distinct competitive contexts—including both national and international tournaments. The present study is the first to systematically investigate how the stage and level of competition affect in-match physical outputs in elite CP football players using objective data from wearable IMUs. This novel approach allows for a deeper understanding of performance variability across the competitive spectrum and can inform tailored training interventions that reflect the true demands of top-level play. Given the increasing professionalization of CP football, the implementation of evidence-based classification systems, and the upcoming international competitions, this research is both timely and necessary to support athlete preparation, classification accuracy, and competitive equity in para-sport.

The effects of participating in different competitions—or in distinct phases of a single competition where competitive demands may vary—remain unexplored in the current literature, representing a relevant gap in understanding how match context influences performance across the competitive spectrum in CP football. To address this gap, the present study aimed to conduct a comparative analysis of in-match physical and technical requirements across different competitive contexts in CP football. Specifically, the aim of this study was to analyze data from IMUs recorded during matches from three different stages of a national league and from an international championship (World Cup), hypothesizing that more demanding competitive contexts will impose greater physical requirements on CP football players.

## 2. Materials and Methods

### 2.1. Design

This study employed a longitudinal observational design with repeated measures, collecting cross-sectional data at multiple time points during the 2023–2024 season. Physical performance variables were recorded by the investigators using wearable inertial measurement technology during official matches across three competitive contexts: the Regular Phase (42 matches, 7 matchdays, from January to April 2024, Spain) and Final Phase (14 matches, divided into Consolation and Playoffs, from May to June 2024, Spain) of the Spanish National 7-a-side Cerebral Palsy Football League (N7FL), and the International Federation of Cerebral Palsy Football (IFCPF) World Cup (6 matches, November 2024, Spain). All matches were played on artificial turf pitches under standardized competition conditions, in accordance with the official IFCPF rules for CP football. These regulations ensure consistency across tournaments regarding pitch dimensions, match duration, number of players, and game structure. As a result, environmental and organizational differences between competitions were minimal. For each player, data were averaged per match within each competitive context to compare the physical requirements.

### 2.2. Participants

In Sample 1, a total of 79 male CP football players were included. The primary inclusion criterion was participation as a starting outfield player in at least one match in each competitive context. Only starting outfield players were included to ensure consistent match exposure and to avoid variability caused by limited playing time. Substitutes may display disproportionately high-performance values due to short, high-intensity efforts performed without accumulated fatigue, which could bias the results. In contrast, starting players experience more sustained and representative match demands, making them more suitable for comparative analysis across competitive contexts. Goalkeepers were excluded due to the clear distinct physical demands of their role. Player distribution across contexts was as follows: Regular Phase (n = 68; FT1: 8, FT2: 51, FT3: 9), Consolation Phase (n = 25; FT1: 5, FT2: 16, FT3: 4), Playoffs (n = 26; FT1: 2, FT2: 21, FT3: 3), and IFCPF World Cup (n = 12; FT1: 1, FT2: 10, FT3: 1).

Sample 2 comprised a subgroup of 10 players from Sample 1 who participated in all 3 competitive contexts (Regular Phase, Playoffs, and World Cup) and was monitored across all matches. All were outfield players, classified as FT2.

All participants provided written informed consent in accordance with the Declaration of Helsinki. The study was approved by the ethics committee of the holding institution of the researchers (REF: ADH.DES.IPG.JFM.24).

### 2.3. Procedures

In-match physical requirements were monitored using a wearable inertial measurement unit (OLI, Oliver IMU^®^, Barcelona, Spain) with a sampling frequency of 10 Hz. Devices were securely attached to the dominant leg of each outfield player at the start of each match. Unlike conventional tracking systems, the OLI’s placement enabled measurement of both the frequency and velocity of ball contacts. The validity and reliability of this tracking system has been reported previously [15,16].

All variables, except maximal velocity and maximum striking force, were normalized to total playing time (expressed as per-minute values). This method, widely used in team sports performance analysis, reflects the relative intensity of match demands and minimizes bias from unequal exposure. Although it may overestimate performance in players with short playing time—especially when high-intensity actions occur in brief periods without accumulated fatigue—this limitation was mitigated by including only starting outfield players, who consistently accumulated substantial playing time across matches. to account for variations in match duration. The physical variables measured are detailed below.

In-match physical variables:

- Maximal velocity (km·h^−1^): The highest movement speed reached by the player during the match.
- Total distance (m·min^−1^): The cumulative distance covered by the player throughout the match.
- Walking distance (m·min^−1^): Distance covered at a walking pace (<6 km·h^−1^).
- Low-intensity running (LI running, m·min^−1^): Distance covered at speeds between 6–12 km·h^−1^.
- Moderate-intensity running (MI running, m·min^−1^): Distance covered at speeds between >12–18 km·h^−1^.
- High-intensity running (HI running, m·min^−1^): Distance covered at speeds exceeding 18 km·h^−1^.
- Dribbling distance (m·min^−1^): Total distance covered while maintaining control of the ball through dribbling.
- Striking force (km·h^−1^): The maximum velocity achieved during a ball strike.
- Total ball contacts (n·min^−1^): The overall number of ball touches recorded during the match.
- Low-intensity ball contacts (n·min^−1^): Ball contacts executed with an ankle velocity <11 m·s^−1^ but an acceleration >20 G.
- Moderate-intensity ball contacts (n·min^−1^): Ball contacts performed with an ankle velocity of 11–15 m·s^−1^.
- High-intensity ball contacts (n·min^−1^): Ball contacts occurring at an ankle velocity greater than 15 m·s^−1^.
- Moderate-intensity accelerations (MI accelerations, m·min^−1^): Distance covered while accelerating between >2 and 3 m·s^−2^.
- High-intensity accelerations (HI accelerations, m·min^−1^): Distance covered while accelerating beyond 3 m·s^−2^.
- Moderate-intensity decelerations (MI decelerations, m·min^−1^): Distance covered while decelerating between <−2 and −3 m·s^−2^.
- High-intensity decelerations (HI decelerations, m·min^−1^): Distance covered while decelerating beyond <−3 m·s^−2^.

### 2.4. Statistical Analysis

Data normality was assessed using the Shapiro–Wilk test. The homogeneity of variances was assessed using Levene’s test. For Sample 1, one-way analyses of variance (ANOVAs) were conducted to compare in-match physical demands across competitive contexts, with Bonferroni post-hoc tests for pairwise comparisons. For Sample 2, repeated-measures ANOVAs were performed, applying the Greenhouse–Geisser correction when sphericity was violated. Effect sizes were calculated as partial eta squared (ηp^2^), interpreted as small (0.01–0.05), moderate (0.06–0.13), large (0.13–0.25), or very large (>0.25) [17]. Data were analyzed using Microsoft Excel v17.0 (Microsoft, Seattle, WA, USA) and JASP (version 0.13, Amsterdam, The Netherlands). Statistical significance was set at *p* < 0.05.

## 3. Results

Shapiro–Wilk test showed a normal distribution for all the variables according to the different competitive contexts (0.97–0.90; *p* > 0.05). Levene’s test indicated a violation of the assumption of homogeneity of some variances. In cases where this assumption was violated, Welch’s ANOVA correction was applied to ensure the robustness of the analysis.

**Figure 1:**
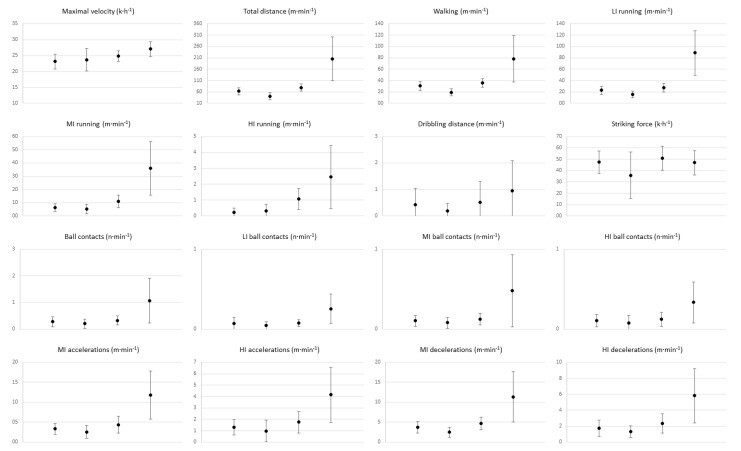
Comparison of in-match physical requirements for Sample 1 across (1) Regular phase, (2) Consolation, (3) Playoffs, and (4) World Cup contexts.

**Figure 2:**
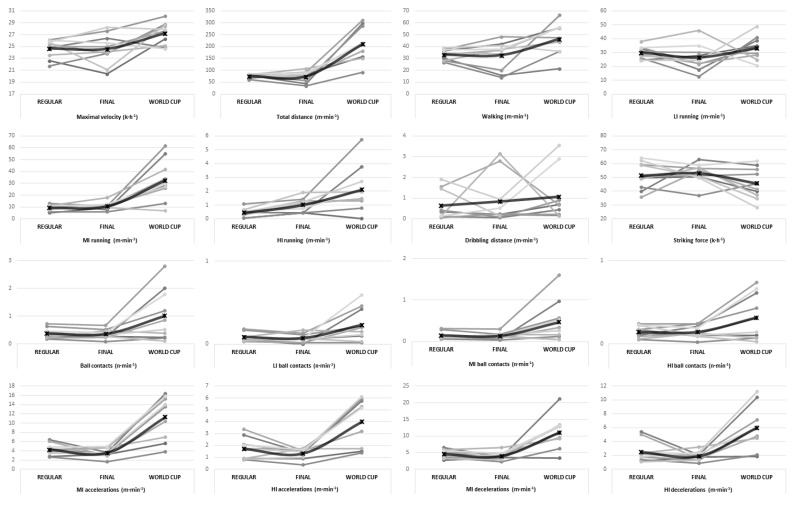
Individual (in grey lines) and average (in black line) player responses for physical and technical requirements across competitive contexts for Sample 2.

The ANOVAs revealed significant differences in the physical requirements of players depending on the competitive context. Overall, the World Cup (international) context showed the highest physical demanding context compared to the Regular Phase, Consolation Phase, and Playoffs of the Spanish N7FL (Table 1; Figure 1). Players reached a higher maximum velocity in the World Cup than in other contexts (Sample 1: F = 16.08, *p* < 0.01, ηp^2^ = 0.28; Sample 2: F = 6.87, *p* = 0.01, ηp^2^ = 0.43) (Table 1 and Table 2; Figure 1 and Figure 2). Similarly, players covered a greater total distance in the World Cup than in any other competitive context, with a very large effect size in both samples (Sample 1: F = 78.98, *p* < 0.001, ηp^2^ = 0.65; Sample 2: F = 16.82, *p* < 0.001, ηp^2^ = 0.65). The Playoffs also required significantly greater total distance covered compared to the Regular and Consolation Phases (*p* < 0.001). A similar pattern was observed for running intensity. The distance covered while walking and at low, moderate, and high intensities was significantly higher in the World Cup context (F = 18.63–68.74, *p* < 0.001, ηp^2^ between 0.31 and 0.68, very large effect size). The Playoffs showed intermediate values, while the Consolation Phase exhibited the lowest physical requirements. Notably, the distance covered at high intensity during the World Cup was more than twice that of the Regular and Consolation Phases (Sample 1: F = 39.20, *p* < 0.001, ηp^2^ = 0.48; Sample 2: F = 10.91, *p* = 0.01, ηp^2^ = 0.55).

The player’s requirements for accelerations and decelerations were considerably higher in the World Cup context, with significant differences across all comparisons. Both moderate- and high-intensity accelerations and decelerations showed markedly greater values in this context compared to the Regular Phase, Consolation Phase, and Playoffs for both samples (Sample 1: F = 28.86–49.05, *p* < 0.001, ηp^2^ = 0.41–0.54; Sample 2: F = 9.52–17.67, *p* < 0.001, ηp^2^ = 0.51–0.66).

The total number of ball contacts was significantly higher in the World Cup context compared to all other phases (*p* < 0.001, ηp^2^ between 0.23 and 0.38, very large effect size). This trend was also observed in low-, moderate-, and high-intensity ball contacts, although the differences were less pronounced and statistically non-significant in Sample 2. Similarly, the distance covered while dribbling and striking force showed significant but moderate differences between competitive contexts in Sample 1 and these differences were non-significant (*p* > 0.05) for Sample 2.

## 4. Discussion

The present study examined the physical and technical requirements of CP football across varying competitive contexts: the Regular Phase, Consolation Phase, and Playoffs of the national CP football league, and the IFCPF World Cup. The results demonstrate that competition level significantly influences in-match player’s physical requirements, with the World Cup imposing the greatest values: higher maximal velocities, greater total and high-intensity running distances, more frequent accelerations and decelerations, and increased ball contacts compared to national-level contexts. The Playoffs also exhibited elevated demands relative to the Regular and Consolation Phases, with the latter showing the lowest physical and technical requirements. These findings, consistent across both a broad sample (n = 79) and a longitudinal subgroup (n = 10), suggest that international competitions necessitate enhanced physical and technical preparation, likely driven by heightened competitive intensity and tactical complexity.

The influence of competitive level on players’ physical requirements remains a debated topic in mainstream football. Some authors have suggested that players competing at lower competitive levels may cover greater distances—particularly at high intensity—possibly due to lower technical and tactical skills [10]. Specifically, no significant differences in distance covered at various intensities have been found between national and international competitions in some studies [12]. However, other research has shown that players at higher competitive levels perform more high-intensity runs and sprints, linking greater competition level with greater physical requirements during matches [11], which aligns with the present results. This trend has also been observed in women’s football [18]. However, the interaction with other contextual factors—such as playing position, match status, or game time—may better explain variations in physical requirements than competition level alone [10,11,12].

The present study showed that running distances increased with competitive level in CP football, with the World Cup requiring significantly greater distances at low, moderate, and high intensities, along with higher maximal velocities and more frequent accelerations and decelerations. The Playoffs displayed intermediate physical demands, while the Consolation Phase consistently showed the lowest physical and technical requirements. Although one might attribute this pattern to differences in players’ functional capacities, no differences in functional class distribution were observed across contexts, and the consistent patterns seen in Sample 2 further suggest that functional classification alone does not explain the reduced demands. Instead, contextual factors such as lower match intensity, diminished competitive stakes, greater disparities in team performance, and potentially reduced player motivation likely contributed to these differences. These findings reinforce the idea that player performance in team sports is shaped not only by physical fitness but also by situational and contextual factors. Previous studies in CP football have similarly linked greater in-match physical requirements to more demanding competitive contexts, whether due to tournament stage or strength of opposition [5]. This is consistent with the present findings, where running distances increased in the more demanding phases of the same tournament (Consolation < Regular < Playoffs) and in matches involving higher-level opposition (national playoffs or international competitions).

One possible explanation for the higher physical requirements observed during the Playoffs and World Cup is the superior physical performance of players involved in these stages compared to those in the Regular and Consolation phases. This aligns with findings from mainstream football, where higher-level players exhibit greater in-match physical demands and superior performance in physical tests [11], as well as evidence from CP football showing that international players outperform non-selected counterparts [13]. Additionally, field-based test results in CP football correlate positively with in-match physical requirements [14]. However, in Sample 2, variables such as maximal velocity, total distance, and moderate- and high-intensity running, accelerations, and decelerations remained significantly higher in international contexts, despite involving the same players. This indicates that, beyond individual physical capacities, the competitive context itself (i.e., international competition) independently elevates physical demands, likely driven by factors such as game pace, pressing strategies, and tactical formations that increase the frequency and intensity of sprinting actions [19].

Similarly, the increased frequency of accelerations and decelerations observed during international competition in the present study indicates a more dynamic style of play, marked by rapid changes of speed and direction, consistent with the greater tactical complexity and performance standards at this level. This trend aligns with previous findings in both able-bodied and CP football [5,18,20]. The heightened demand for such actions in the World Cup context highlights the greater neuromuscular and metabolic demands placed on players at the elite level, underscoring the need for training programs that specifically target acceleration and deceleration capacities for international competition.

In addition to locomotor variables, the present findings highlight differences in technical actions, such as ball contacts and dribbling distance. Players recorded significantly more ball contacts per minute in the World Cup, particularly in Sample 1, suggesting more continuous involvement in gameplay driven by the greater technical and tactical demands of international competition. This aligns with findings in elite mainstream football, where higher-level players perform more high-quality technical actions. For example, Bradley et al. [10] reported more touches per possession among Premier League players compared to lower-level counterparts, while Dellal et al. [21] showed that professional players not only execute more high-intensity actions but also achieve superior technical performance, with more successful passes and fewer ball losses during small-sided games.

The elevated technical requirements observed in CP football at the international level reinforce that, despite neurological impairments, players adapt to the game’s pace and complexity similarly to those in mainstream football. While differences in ball contacts were statistically significant in Sample 1, they were less pronounced and non-significant in Sample 2, where the same players competed across contexts. This suggests that individual playing styles or tactical roles may have influenced the results. Moreover, the absence of significant differences in Sample 2 indicates that player characteristics—particularly higher technical proficiency among those in more competitive settings (Sample 1)—may impact technical requirements more than the competitive context itself. Similarly, dribbling distance and striking force showed only moderate differences across contexts in Sample 1 and were non-significant in Sample 2, suggesting that technical performance may depend more on individual skill level and match strategy than on competition level alone.

The results of this study provide valuable guidance for the preparation of CP football players across different competitive contexts. Given the substantially higher physical and technical requirements observed in international matches—particularly in sprinting, high-intensity running, frequent accelerations and decelerations, and increased ball interactions—training programs should be specifically designed to progressively develop these capacities. Players should be regularly exposed to high-intensity locomotor actions through drills that replicate the speed and directional changes of elite-level play. The elevated technical involvement observed in international contexts, especially in terms of ball contacts, underscores the need to train technical execution under pressure and fatigue. Small-sided games and positional drills that simulate match conditions and involve high cognitive and neuromotor demands (e.g., decision-making, dribbling, and striking under time and space constraints) may be particularly effective.

The variation in physical requirements across national competition phases (Consolation < Regular < Playoffs) further suggests that not all domestic matches offer the same stimulus. Therefore, coaching staff should manage training loads accordingly throughout the season and consider supplemental, individualized conditioning for players preparing for international competition—especially those coming from less demanding domestic contexts. Specific training strategies should include high-intensity drills focused on repeated accelerations and decelerations, change of direction exercises, and small-sided games that promote frequent technical interactions under pressure and fatigue. In particular, integrating drills that replicate the cognitive and physical demands of international matches—such as rapid transitions, pressing actions, and dynamic ball involvement—can help better prepare players for the heightened requirements of elite competition. Overall, these findings support a comprehensive training approach in CP football, where physical, technical, tactical, and cognitive components are integrated to meet the requirements of increasingly competitive match environments. Data obtained from IMUs in matches between teams of similar skill levels can help in the classification process of players with neurological conditions.

This study presents several limitations that should be acknowledged. The absence of tactical information—such as team formations, possession durations, or other contextual match variables—limits the interpretation of how strategic factors may have influenced player physical requirements across competitive contexts. Although a subgroup of players was monitored longitudinally across all three competitive contexts, the small sample size (n = 10) reduces the statistical power and limits the generalizability of intra-individual comparisons. As a result, the analysis may have been insufficient to detect subtle differences across competitive contexts. A post-hoc power analysis indicated that, with this sample size, the statistical power to detect medium-sized effects was approximately 76%, whereas smaller effects may have remained undetected. Additionally, certain potential confounding variables—such as players’ field positions, effective playing time, and fatigue status between phases—were not controlled for and may have influenced the observed performance metrics. Furthermore, given the large number of variables analyzed, no global correction for multiple comparisons across all variables was applied, which may increase the risk of type I error and should be considered when interpreting the results. Future research should aim to integrate physical performance monitoring with contextual and tactical analyses, including video- or notational-based assessments, to better understand how match strategies influence physical requirements. Finally, considering the heterogeneity of impairments in CP football, future studies should examine whether physical requirements differ across sport classes (FT1, FT2, FT3) within the same competitive context, as this may have implications for classification processes, talent identification, and tactical role assignment.

## 5. Conclusions

This study demonstrated that the physical and technical requirements of CP football increase with the competitive level. The IFCPF World Cup showed the highest values in total distance, high-intensity running, maximal velocity, accelerations, decelerations, and ball contacts. Playoff matches presented intermediate requirements, while the Consolation Phase consistently recorded the lowest. Importantly, these differences were evident even when the same players participated across contexts, suggesting that the competitive environment itself—rather than just individual player capacity—drives the increased player’s in-match requirements. Contextual factors such as match intensity, tactical complexity, and game dynamics likely could explain these variations. Additionally, technical requirements were greater in more competitive settings, particularly in Sample 1, reinforcing the idea that higher-level matches require greater involvement and precision in gameplay.

## Figures and Tables

**Table 1:** In-match physical requirement differences between competitions from sample 1.

In-Match Physical Requirements	Regular	Consolation	Playoffs	World Cup	F (*p*)	ηp^2^ (Descriptor)
Maximal velocity (k·h^−1^)	23.10 ± 2.33	23.69 ± 3.49	24.80 ± 2.35	27.02 ± 1.68 ^ab^	16.08 (<0.001)	0.28 (very large)
Total distance (m·min^−1^)	61.76 ± 16.43	40.60 ± 15.89 ^a^	78.72 ± 15.72 ^b^	205.31 ± 95.96 ^abc^	78.98 (<0.001)	0.65 (very large)
Walking (m·min^−1^)	30.43 ± 8.10	19.07 ± 6.20 ^a^	35.82 ± 7.58 ^ab^	42.14 ± 15.88 ^ab^	25.00 (<0.001)	0.37 (very large)
LI running (m·min^−1^)	22.75 ± 7.17	15.62 ± 5.78 ^a^	27.49 ± 7.60 ^ab^	32.89 ± 10.65 ^ab^	18.63 (<0.001)	0.31 (very large)
MI running (m·min^−1^)	6.40 ± 2.97	5.24 ± 3.66	10.95 ± 4.64 ^ab^	36.08 ± 20.17 ^abc^	68.74 (<0.001)	0.62 (very large)
HI running (m·min^−1^)	0.23 ± 0.25	0.33 ± 0.41	1.07 ± 0.67 ^ab^	2.46 ± 1.99 ^abc^	39.20 (<0.001)	0.48 (very large)
Dribbling distance (m·min^−1^)	0.43 ± 0.60	0.19 ± 0.29	0.52 ± 0.80	0.94 ± 1.15 ^b^	3.57 (0.02)	0.08 (moderate)
Striking force (k·h^−1^)	47.39 ± 9.92	41.00 ± 16.17	50.76 ± 10.56 ^b^	46.97 ± 10.71	2.86 (0.04)	0.07 (moderate)
Ball contacts (n min^−1^)	0.28 ± 0.19	0.20 ± 0.18	0.32 ± 0.17	1.07 ± 0.84 ^abc^	25.45 (<0.001)	0.38 (very large)
LI ball contacts (n·min^−1^)	0.07 ± 0.08	0.05 ± 0.05	0.08 ± 0.04	0.25 ± 0.19 ^abc^	18.65 (<0.001)	0.31 (very large)
MI ball contacts (n·min^−1^)	0.10 ± 0.07	0.08 ± 0.07	0.13 ± 0.07	0.48 ± 0.45 ^abc^	23.52 (<0.001)	0.36 (very large)
HI ball contacts (n·min^−1^)	0.11 ± 0.08	0.08 ± 0.09	0.12 ± 0.09	0.33 ± 0.26 ^abc^	16.70 (<0.001)	0.28 (very large)
MI accelerations (m·min^−1^)	1.74 ± 1.04	1.31 ± 0.75	2.35 ± 1.20 ^b^	5.82 ± 3.40 ^abc^	32.63 (<0.001)	0.44 (very large)
HI accelerations (m·min^−1^)	1.28 ± 0.68	0.97 ± 0.96	1.75 ± 0.95 ^b^	4.15 ± 2.43 ^abc^	28.86 (<0.001)	0.41 (very large)
MI decelerations (m·min^−1^)	3.65 ± 1.44	2.44 ± 1.27	4.71 ± 1.56 ^b^	11.31 ± 6.33 ^abc^	44.13 (<0.001)	0.51 (very large)
HI decelerations (m·min^−1^)	3.30 ± 1.35	2.56 ± 1.57	4.34 ± 2.14 ^b^	11.76 ± 6.04 ^abc^	49.05 (<0.001)	0.54 (very large)

LI: low intensity; MI: moderate intensity; HI: high intensity. ^a^ statistically different of Regular; ^b^ statistically different of Consolation; ^c^ statistically different of Playoffs.

**Table 2:** In-match physical requirement differences between competitions from sample 2.

In-Match Physical Requirements	Regular	Playoffs	World Cup	F (*p*)	ηp^2^
Maximal velocity (k·h^−1^)	24.66 ± 1.53	24.61 ± 2.53	27.24 ± 1.91 ^a^	6.87 (0.01)	0.43 (very large)
Total distance (m·min^−1^)	75.63 ± 7.25	73.94 ± 22.53	193.48 ± 90.94 ^ab^	16.82 (<0.001)	0.65 (very large)
Walking (m·min^−1^)	33.10 ± 4.57	32.74 ± 11.80	42.94 ± 17.18	2.75 (0.09)	0.23 (large)
LI running (m·min^−1^)	29.83 ± 4.46	26.34 ± 9.32	31.10 ± 10.77	0.69 (0.51)	0.07 (moderate)
MI running (m·min^−1^)	9.36 ± 2.50	10.69 ± 3.12	32.23 ± 16.93 ^ab^	19.19 (<0.001)	0.68 (very large)
HI running (m·min^−1^)	0.44 ± 0.30	1.00 ± 0.49 ^a^	2.15 ± 1.57 ^ab^	10.91 (0.01)	0.55 (very large)
Dribbling distance (m·min^−1^)	0.64 ± 0.70	0.84 ± 1.15	0.96 ± 1.23	0.27 (0.77)	0.03 (small)
Striking force (k·h^−1^)	51.17 ± 9.71	52.87 ± 7.02	45.49 ± 11.18	1.90 (0.18)	0.17 (large)
Ball contacts (n min^−1^)	0.38 ± 0.18	0.35 ± 0.17	1.01 ± 0.92	4.67 (0.10) #	0.23 (large)
LI ball contacts (n·min^−1^)	0.08 ± 0.05	0.07 ± 0.05	0.23 ± 0.20	5.28 (0.07) #	0.26 (very large)
MI ball contacts (n·min^−1^)	0.15 ± 0.09	0.14 ± 0.07	0.46 ± 0.49	4.05 (0.13) #	0.20 (large)
HI ball contacts (n·min^−1^)	0.15 ± 0.07	0.14 ± 0.07	0.32 ± 0.27	3.32 (0.19) #	0.17 (large)
MI accelerations (m·min^−1^)	4.20 ± 1.24	3.53 ± 1.01	10.34 ± 5.38 ^ab^	17.67 (<0.001)	0.66 (very large)
HI accelerations (m·min^−1^)	1.72 ± 0.89	1.32 ± 0.44	3.65 ± 2.18 ^ab^	12.33 (<0.001)	0.58 (very large)
MI decelerations (m·min^−1^)	4.49 ± 1.27	3.95 ± 1.21	10.11 ± 5.54 ^ab^	13.65 (<0.001)	0.60 (very large)
HI decelerations (m·min^−1^)	2.44 ± 1.54	1.67 ± 0.66	5.41 ± 3.53 ^ab^	9.52 (0.01)	0.51 (very large)

LI: low intensity; MI: moderate intensity; HI: high intensity. ^a^ statistically different of Regular; ^b^ statistically different of Playoffs. # Greenhouse-Geisser correction for sphericity was applied.

## Data Availability

The datasets generated and analyzed during the current study are not publicly available but may be made available by the corresponding author upon reasonable request. Data will be accessible for a period of three years following the publication of this article. Interested researchers must contact the corresponding author via email with a justified explanation of the intended use of the data. Access will be granted at the discretion of the corresponding author, provided that the request aligns with ethical standards and data protection regulations.
